# SignaLink 2 – a signaling pathway resource with multi-layered regulatory networks

**DOI:** 10.1186/1752-0509-7-7

**Published:** 2013-01-18

**Authors:** Dávid Fazekas, Mihály Koltai, Dénes Türei, Dezső Módos, Máté Pálfy, Zoltán Dúl, Lilian Zsákai, Máté Szalay-Bekő, Katalin Lenti, Illés J Farkas, Tibor Vellai, Péter Csermely, Tamás Korcsmáros

**Affiliations:** 1 Department of Genetics, Eötvös Loránd University, Pázmány P. s. 1C, H-1117, Budapest, Hungary; 2 Statistical and Biological Physics Group of the Hungarian Acad. of Sciences, Pázmány P. s. 1A, H-1117, Budapest, Hungary; 3 Department of Biological Physics, Eötvös Loránd University, Pázmány P. s. 1A, H-1117, Budapest, Hungary; 4 Department of Medical Chemistry, Semmelweis University, PO Box 260, H-1444, Budapest, Hungary; 5 Department of Morphology and Physiology, Semmelweis University, Vas u. 17, H-1088, Budapest, Hungary

**Keywords:** Signal transduction, Signaling network, Regulatory network, Scaffold, miRNA, Transcription factor, Drug discovery, Signaling database

## Abstract

**Background:**

Signaling networks in eukaryotes are made up of upstream and downstream subnetworks. The upstream subnetwork contains the intertwined network of signaling pathways, while the downstream regulatory part contains transcription factors and their binding sites on the DNA as well as microRNAs and their mRNA targets. Currently, most signaling and regulatory databases contain only a subsection of this network, making comprehensive analyses highly time-consuming and dependent on specific data handling expertise. The need for detailed mapping of signaling systems is also supported by the fact that several drug development failures were caused by undiscovered cross-talk or regulatory effects of drug targets. We previously created a uniformly curated signaling pathway resource, SignaLink, to facilitate the analysis of pathway cross-talks. Here, we present SignaLink 2, which significantly extends the coverage and applications of its predecessor.

**Description:**

We developed a novel concept to integrate and utilize different subsections (i.e., layers) of the signaling network. The multi-layered (onion-like) database structure is made up of signaling pathways, their pathway regulators (e.g., scaffold and endocytotic proteins) and modifier enzymes (e.g., phosphatases, ubiquitin ligases), as well as transcriptional and post-transcriptional regulators of all of these components. The user-friendly website allows the interactive exploration of how each signaling protein is regulated. The customizable download page enables the analysis of any user-specified part of the signaling network. Compared to other signaling resources, distinctive features of SignaLink 2 are the following: 1) it involves experimental data not only from humans but from two invertebrate model organisms, *C. elegans* and *D. melanogaster*; 2) combines manual curation with large-scale datasets; 3) provides confidence scores for each interaction; 4) operates a customizable download page with multiple file formats (e.g., BioPAX, Cytoscape, SBML). Non-profit users can access SignaLink 2 free of charge at http://SignaLink.org.

**Conclusions:**

With SignaLink 2 as a single resource, users can effectively analyze signaling pathways, scaffold proteins, modifier enzymes, transcription factors and miRNAs that are important in the regulation of signaling processes. This integrated resource allows the systems-level examination of how cross-talks and signaling flow are regulated, as well as provide data for cross-species comparisons and drug discovery analyses.

## Background

Reliable analyses of signaling pathways need uniform pathway definitions and curation rules applied to all pathways. Accordingly, we previously created SignaLink, a resource containing major signaling pathways of the nematode *Caenorhabditis elegans*, the fruit fly *Drosophila melanogaster* and humans [1]. Specific regulation of signaling flow is essential to ensure the appropriate response of the signaling system for a given input [2]. Signaling flow is determined by the spatial and temporal properties of signaling proteins precisely regulated by cellular processes (e.g., endocytosis, transcription, miRNA regulation) [3]. In addition, signaling components are modulated by proteins having no direct signaling functions, such as scaffold proteins and ubiquitin ligases [4,5].

Despite the complexity of eukaryotic signaling networks, current signaling or regulation-related resources contain only specific parts of such a global signaling network. As a consequence, computational background and expertise in different bioinformatics resources are needed to answer questions about how a signaling pathway is regulated, or how the pathways influence each other through transcription and miRNA-mediated gene silencing. A few studies already combined regulatory and protein-protein interaction networks [6-9], while a new resource, TranscriptomeBrowser, integrates human regulatory networks with phosphorylation reactions [10]. Recently, we developed a systems-level resource of the transcription factor NRF2, containing its transcriptional, post-transcriptional and post-translational modifiers, based on manual curation, *in silico* prediction and existing dataset imports [11].

Taking into consideration the need for a novel arrangement of signaling and regulatory data to examine signaling processes on a systems-level, we present SignaLink 2, a database with multi-layered (onion-like) structure. Our basic aim was to develop an integrated database that helps anyone to understand how cellular signaling pathways and their cross-talks are regulated. To accomplish this goal, SignaLink 2 contains for worms, flies and humans 1) pathway components and cross-talks; 2) interacting proteins that modify or facilitate signaling reactions; and 3) regulatory components (transcription factors and miRNAs) that affect the expression of pathway proteins and their interactors.

## Construction and content

### Compilation of the multi-layered network

**Figure 1:**
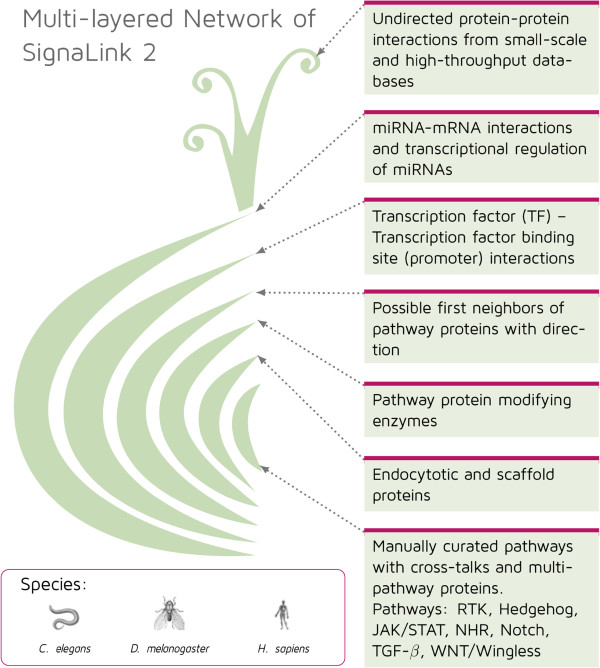
**The multi-layered structure of SignaLink 2.** Layers are from different sources and contain different types of interactions. The image of an onion is used to illustrate this structure. The core of the database contains the interactions between pathway member proteins. In the first layer these proteins are connected with pathway regulators, such as scaffold and endocytotic proteins. The next two layers contain the first neighbor interactors of these proteins. The interactors have a predicted enzymatic effect (second layer) or a known physical interaction (third layer) to the members of the core or the first layer. The fourth and the fifth layers contain the transcription factors (TFs) and miRNA regulators of the already listed proteins, respectively. The fifth layer also contains the TFs of these miRNAs.

We developed an onion-like, multi-layered database structure to integrate and utilize the different subsections (i.e., layers) of the signaling network. The multi-layered structure and content of each layer is illustrated and listed in Figure 1. 

The core of SignaLink 2 contains seven major pathways, which are biochemically and evolutionary defined, and encompass all major developmental signaling mechanisms [12]: RTK (receptor tyrosine kinase), TGF-ß (transforming growth factor beta), WNT/Wingless, Hedgehog, JAK/STAT, Notch and NHR (Nuclear hormone receptor). We note that in the previous version of SignaLink, EGF/MAPK (epidermal growth factor /mitogen-activated protein kinase) and insulin/IGF (insulin growth factor) pathways were defined as separate pathways. In this upgraded and extended version, the RTK pathway contains both pathways and additional related receptors (e.g., VEGFR and FGFR). This grouping is more realistic and in line with evolutionary studies [12]. While earlier in SignaLink the NHR pathway contained only the NHR proteins, it now includes their co-factors, too. The uniform manual curation protocol remained the same as developed and published earlier [1]. Accordingly, we set the pathway boundaries based on expert-written reviews and manual search of the literature. We examined the signaling functions and interactions of the proteins mentioned in the reviews. For each signaling interaction, we listed the PubMed ID of the publication reporting the verifying experiment(s). In addition, we grouped all manually curated signaling pathway proteins to ‘core’ and ‘non-core’ proteins. A ‘core’ protein is essential for transmitting the signal of its pathway, while a ‘non-core’ protein modulates the pathway’s core proteins but not transmit the incoming signal. For a more detailed description on the curation protocol, please see the supplementary material of our earlier publication on SignaLink [1]. The current curation update was closed in April, 2011.

We added two further extensions that can be optionally used. i) With manual curation, we collected scaffold proteins and endocytosis-related proteins and linked them to signaling pathway proteins, based on the scaffold protein list of the Ref. [13] and signaling-related endocytosis reviews, respectively. ii) We extended the number of transcription factors (TFs) in the database from 243 to 586 by connecting additional TFs to already curated TFs, based on protein-protein interaction (PPI) data from WI8, DroID, HPRD and BioGRID databases [14-17].

Next, using the ELM server [18], we searched for enzymes (i.e., phosphatases, ubiquitin-ligases, peptidases, etc.) that can directly modify signaling components involved in SignaLink 2. We then searched for other proteins previously not known to be as signaling-related ones, but having interaction with a component already included. For this, we used the same PPI resources as for the TF-network. Based on the algorithms of the Ref. [19], we predicted directions for the PPIs.

Finally, we identified underlying transcriptional and post-transcriptional regulatory networks that control the expression of signaling components and their interactors. TF–TF binding site interactions were used to list transcriptional connections between TFs and genes encoding signaling components or pathway interactors. We downloaded experimental and predicted data from the EdgeDB, REDFly, DroID, ABS, JASPAR, HTRIdb, OregAnno, ENCODE and PAZAR databases [15,20-27]. We also included two types of miRNA network data: i) miRNA–mRNA interactions from miRBase, TarBase, Miranda, TargetScan and miRecords [28-32], and ii) TFs of these miRNAs from PutMir, TransMir and ENCODE [27,33,34]. For each regulatory interaction, binding scores were calculated based on position matrix values or inserted from the original sources.

### Integrating the sources and quality control

**Figure 2:**
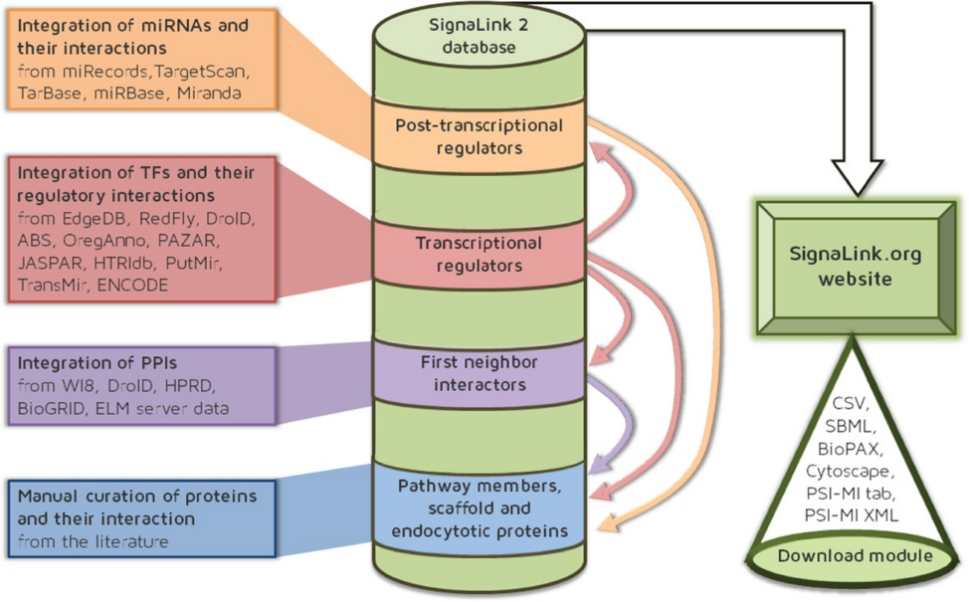
**The construction and structure of SignaLink 2.** First, with manual curation we updated previous pathway data, extended the set of pathway proteins and included information on scaffold and endocytotic proteins. Then, we integrated the protein-protein interactions affecting proteins in the manually curated core of SignaLink 2. With this step we also imported first neighbor proteins of the pathway member and pathway regulator proteins. Then, we integrated the transcription factors (TF) that regulate all these proteins. Finally, we integrated miRNAs and their interactions with the mRNAs of all the already inserted proteins. We also added the TFs of the imported miRNAs.

The core of SignaLink 2 contains the interactions of pathway member proteins and their regulators (endocytotic and scaffold proteins). This data is derived from manual curation of the literature, and has been entered into the database directly. All the other layers of the database were acquired from external resources. To integrate these resources with the core of SignaLink 2 and with each other, we used the original accession numbers of the imported databases (i.e., Entrez Gene ID, NCBI Gene ID, Gene symbols, Ensembl ID, WormBase ID, FlyBase ID, etc.) and mapped them to UniProt Primary IDs using the UniProt Mapping Service [35]. The integration process and the structure of SignaLink 2 are shown in Figure 2. The final number of proteins and interactions in each layer is listed in Table 1.

**Table 1:** Detailed statistics of SignaLink 2

**Species**	***C. elegans***		***D. melanogaster***		***H. sapiens***	
**Layers**	**Nodes**	**Edges**	**Nodes**	**Edges**	**Nodes**	**Edges**
**Pathway members**	198	253	210	260	1,150	1,640
**Pathway regulators**	0	0	0	0	751	2,122
**Post-translational modifiers**	916	3,072	1,713	6,896	4,682	82,852
**Directed protein-protein interactors**	49	47	128	171	951	3,252
**Undirected protein-protein interactors**	100	245	166	496	1,387	6,086
**TF regulators**	152	187	998	16,319	2,585	30,736
**miRNA regulators**	806	9,658	939	5,308	2,844	245,857
**TFs of miRNAs**	25	19	0	0	716	5,209

SignaLink 2 contains different amounts of proteins/miRNAs in each layer in each species, according to the data from the literature and external sources. See the main text for details on the layers and sources. For *C. elegans* and *D. melanogaster*, no data was available for pathway regulator scaffold proteins and for TFs that regulate miRNAs.

**Figure 3:**
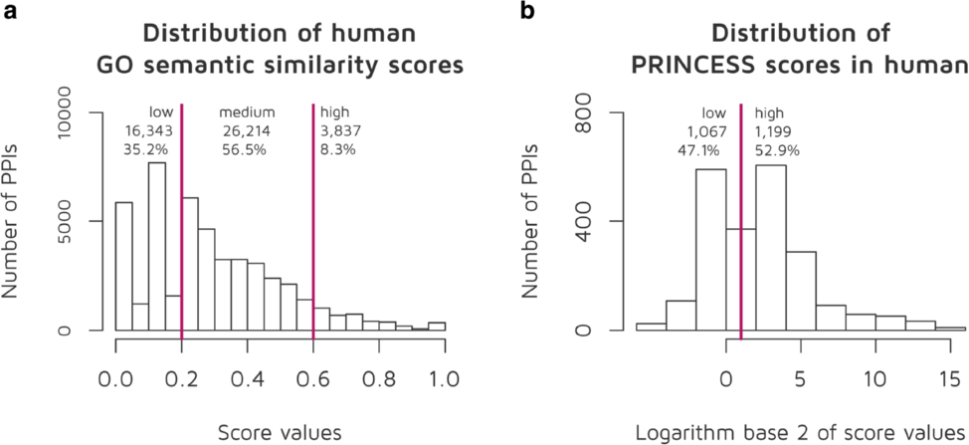
**Confidence scores for human protein-protein interactions (PPIs). a**) Confidence scores calculated based on a GO semantic similarity score [36]. We determined cut-off scores at 0.2 and 0.6 to divide low-, medium- and high-confidence PPIs. **b**) For PPIs in humans where detailed information was available for the interacting proteins, we also calculated another confidence score with the PRINCESS PPI-evaluation tool [37]. By default, PRINCESS sets it cut-off score at 2.0. More than the half (52.9%) of the evaluated PPIs were above this value.

For every data source containing integrated data sets that is interactions collected with different methods, quality control is highly important. For each PPI, we calculated a confidence score based on a GO semantic similarity score [36] (Figure 3a). By performing a ROC curve analysis, we determined two cut-off scores to get three confidence categories. We set the lower cut-off score to 0.2 to divide low- and medium-confidence PPIs. At this cut-off score the false negative rate (FN/N) was 0.058 (i.e. 5.8% of the PPIs were false negatives), and the true positive rate (TP/P) was 0.42 (i.e., 42% of the experimentally known PPIs were true positives). A second cut-off score was also calculated to distinguish between medium and high-confidence PPIs. This cut-off was set to 0.6 with 0.8 true positive rate and 0.23 false negative rate. Applying these cut-off scores we found that, for example for humans, SignaLink 2 contains 16,343 low-confidence PPIs (35.2%), 26,214 medium-confidence PPIs (56.5%) and 3,837 (8.3%) high-confidence PPIs (Figure 3a). Thus, nearly the two-third of the PPIs in SignaLink 2 has medium or high-confidence scores. Note the high number of high-confidence PPIs, which possibly resulted from the common signaling-related functions of the interacting proteins. For PPIs in humans, we also evaluated the interactions with the PRINCESS PPI-evaluation tool [37] (Figure 3b). Calculation of the PRINCESS score involves multiple data types, thus, it can be calculated only for a limited set of interacting proteins for which these information is available. Consequently, we could calculate PRINCESS scores for 2,266 PPIs. Using 2.0 as the default cut-off score of PRINCESS [37], we found 1,067 low-confidence PPIs (47.1%) and 1,199 high-confidence PPIs (52.9%) (Figure 3b). Most of the high-confidence PPIs were originally found by the manual curation of SignaLink 2. We note that PRINCESS score highly depends on the quality of the available information on the interacting proteins. Thus, upon more information will be available, the number of PPIs above the cut-off may increase. We also note that users can optionally decide to set other cut-off scores or simply download the PPIs without a cut-off filter. We note that we could calculate confidence scores for an average of 58% interactions (74,152 PPI in the three species, from which 61,380 PPIs were found in humans). For the remaining interactions the GO annotations of the interacting protein pairs were not known. Thus, SignaLink 2 could contain false positive interactions. Applying confidence scores, checking the literature references listed in SignaLink or using other evaluation tools could help the users to filter the most probable interactions for their analysis. In the case of other binding interactions (e.g., TF-TFBS or mRNA-miRNA), we used the original scores of the source. Distinction of predicted and experimentally verified interactions is also important. Thus, we grouped all the integrated sources accordingly, and implemented an easy selection option at the download page, where users can decide the type of interactions (i.e., original sources) to be included. Furthermore, all scores and details can be customized by advanced users (for details, see the *Utility* section), allowing a personalized level of quality and confidence to be set.

We also note that all experimental interactions in SignaLink either collected by manual curation or integration of other sources were coming from various cell types and experimental conditions. Therefore, interactions in SignaLink 2 are the sum of many possible interactions but not all of these interactions can happen at the same time or same place. Users can integrate cell localization and tissue expression data to the networks of SignaLink 2 to filter compartment- or tissue-specific interactions, respectively. We plan to include such data types in the next version of SignaLink.

### Database structure

**Figure 4:**
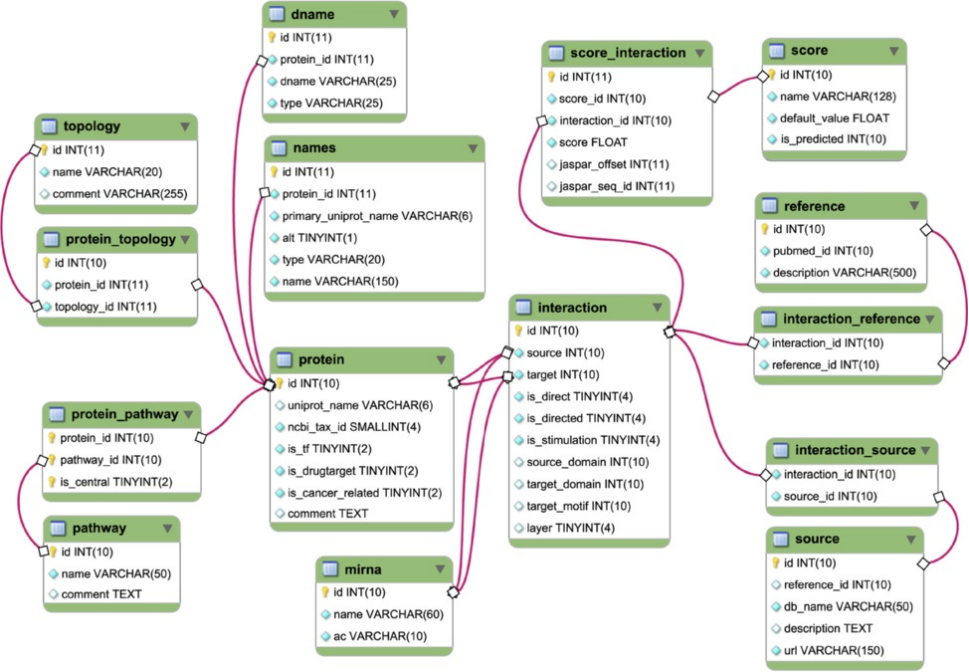
**The SQL database scheme of SignaLink 2.** The core of the database is the interaction table. Interactions can be between proteins or one protein and one miRNA. Each record in the protein or mirna tables represents one real protein or miRNA, and one real entity represented only by one record. There can be more than one interaction between two nodes, but only one within each layer. Within layers, interactions have sources (what databases are they originated from) and reference annotations (what publications do contain information about them) Interaction_source table connects interaction and source tables, while interaction_reference table connects interaction and reference tables. Each interaction has the attributes whether it is direct or indirect (*interaction.is_direct*), directed or undirected (*interaction.is_directed*), stimulatory or inhibitory (*interaction.is_stimulation*) stored in the interaction table. The interaction table is connected to the score_interaction table*,* in which binding and confidence scores are stores. Other supplementary tables contain names and IDs of proteins. See the main text for details.

SignaLink 2 stores data in a MySQL database (for the database schema, see Figure 4), where each record in the protein or miRNA tables represents one real protein or miRNA, respectively, and one real entity is represented only by one record. Proteins might belong to pathways (stored in the protein_pathway table) and have topological attributes (stored in the protein_topology table) such as ligand, receptor, mediator, co-factor, transcription factor, scaffold, endocytosis-related. Within layers, each interaction has one or more sources. For each interaction we list the original database(s) from which it was integrated into SignaLink 2. These database sources are listed in the source table*,* where each source has an attribute whether the source contains *experimentally verified* or *predicted* interactions. In addition, each interaction has one or more literature references (i.e., original publication(s) about the interaction, retrieved from the integrated databases). References are listed in the reference table. In the interaction table, each interaction also has attributes whether it is *direct* or *indirect*, *directed* or *undirected*, *stimulatory* or *inhibitory*. For example, direct interaction occurs between a kinase and its phosphorylation target; and we mean indirect interaction between a transcription factor and its target gene, or between two proteins that are members of a complex but no evidence is known that they directly bind to each other. All literature-curated interactions in SignaLink 2 are directed. For the protein-protein interactions retrieved from external resources, we predicted a single direction. Thus, these interactions are also listed as *directed*, while the remaining PPIs for which we could not determine the direction are listed as *undirected*. Interactions may have *stimulatory* or *inhibitory* effect. Where we could not determine the effect from the original publication or sources, we mention "*stimulatory or inhibitory interaction*". An interaction may have multiple scores, which are stored at the interaction_score table. This table contains the original scores from ELM Structure Filter, TarBase, miRecords, miRanda, PicTar, TargetScan, PutMir and JASPAR as well as the confidence scores we generated with a GO Semantic Similarity-based method and with the PRINCESS protein interaction evaluation tool [36,37].

## Utility

### User-friendly web interface

The webpage processes data with PHP on the server side, and jQuery on the client side, providing a great user experience in all standard compliant browsers. To display interactive networks, we use the Cytoscape Web plugin [38]. The search method performs partial match on multiple types of names and database IDs, finding the proteins and miRNAs matching the text typed in by the user. The search field helps the user in autocompleting the text while typing.

**Figure 5:**
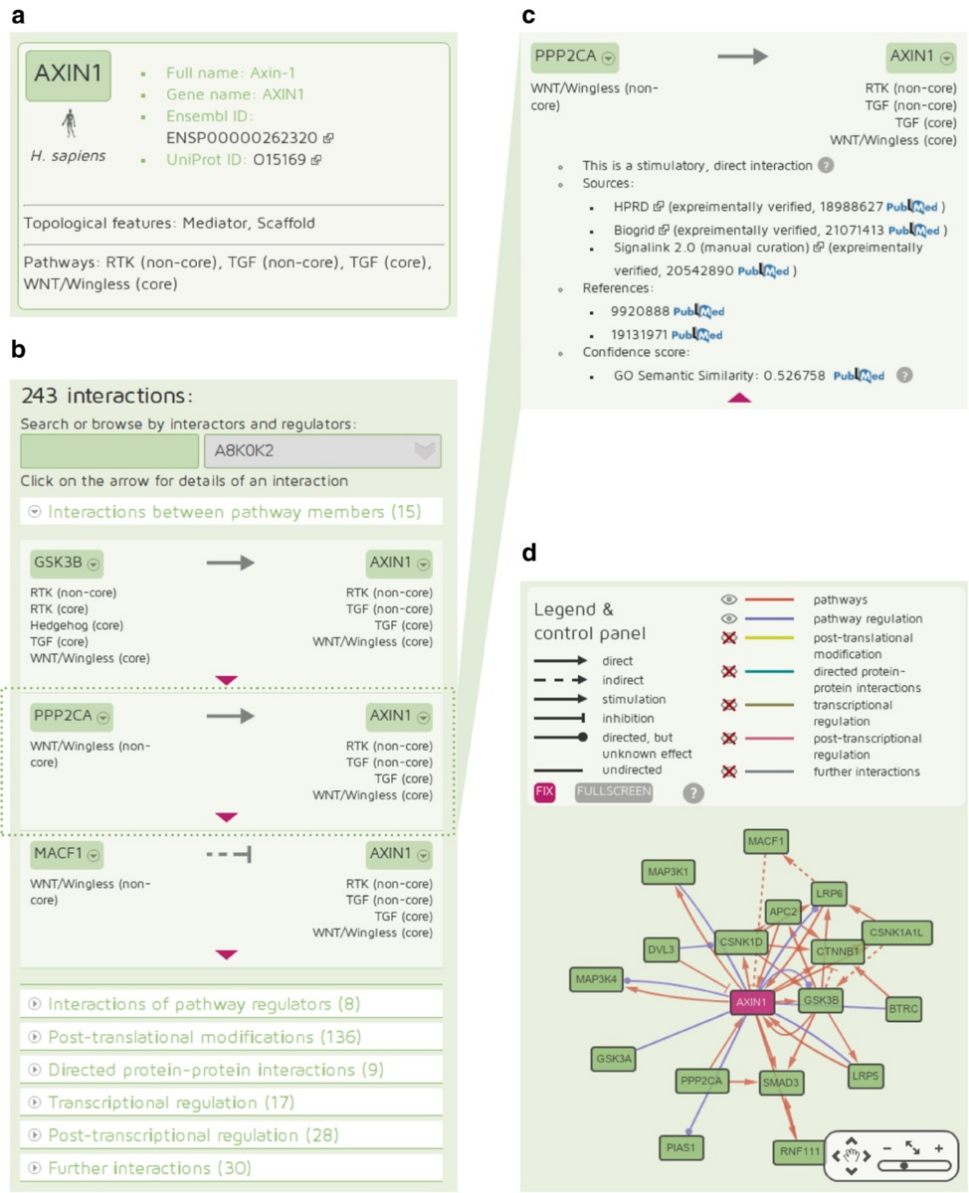
**Functionality of the protein datasheet at the SignaLink.org website. a**) This box contains basic information about the protein, AXIN1: references to other databases, topological features and pathway memberships. **b**) The protein datasheet lists all interactions of AXIN1, grouped by layers. Expanding one layer, users are able to browse the list of interactions. **c**) In this view the properties of the interactions (direction, direct or indirect, stimulatory or inhibitory) are visible. More details (database sources of the interaction, literature references and scores) can be accessed by one click. **d**) An interactive network of first neighbors is available, visualized by the CytoscapeWeb plugin [38].

The protein datasheets of the website display all information stored in SignaLink 2 about the queried protein. In Figure 5, the protein datasheet of AXIN1 is shown. (AXIN1 was used as an example to illustrate the usage of the website, described in detail in the *Discussion* section.) On the top of a protein datasheet basic information is shown: name, species-specific database (Wormbase, Flybase, ENSEMBL) IDs and UniProt accessions. All of these IDs are hyperlinked to the corresponding website to help discovering further details. In the case of pathway member proteins, basic information also includes pathway memberships and topological properties. On the protein datasheet, users can interactively explore the interactions of the queried protein both with a list, where interactions are grouped by layers, and with an interactive network visualization of these interactions. In both cases, the user can easily obtain information on each interaction, including the type of the interaction (direct/indirect, directed/undirected, stimulatory/inhibitory, predicted/experimentally verified), interaction scores from the original source (if applicable) and confidence scores generated by SigaLink 2. The evidence for the interaction is shown with PubMed links to the external source from where we integrated the given interaction, and PubMed links to the original paper used as a reference. The information originated from different integrated resources is easily comparable, and bi-directional relations also have good visibility. The URLs of the protein datasheets are constructed simply from the proteins’ UniProt IDs, with a scheme similar to that of UniProt, e.g., http://signalink.org/protein/P00533. In addition, all protein datasheets can be accessed with gene names or species-specific database IDs allowing the easy linking of these datasheets from other webpages or resources (e.g., http://signalink.org/protein/sma-3, http://signalink.org/protein/FBgn0004859). We have included a comprehensive FAQ site and inserted short popup help-boxes in most of the web pages to support the users.

### Download options

The entire database is available as a MySQL dump file. Alternatively, we developed a BioMART-like customizable download page, where users can easily select and combine the species, pathways, layers and the file format of download. The customized subnetworks can be downloaded in various formats: CSV, BioPAX, SBML, PSI-MI tab or PSI-MI XML and in a Cytoscape CYS file. Data can be compressed by GNU zip or zip. After selecting the details of the download, for advanced users, we offer additional customization where within each layer the different source databases can be filtered by score values, or even excluded. A general switch is also available to exclude all predicted interactions.

All user-specified download options are automatically transformed to MySQL queries. For each download, we generate a URL, where users can access the data for 14 days. Optionally, users can provide their e-mail addresses to which files smaller than 10 MB will be e-mailed. We have developed a download module (written in Python), to manage the queries and to convert the result of the queries to user-specified file formats. In conclusion, SignaLink 2 serves as an integrated signaling resource where the origin, type and confidence level of each interaction are clearly listed, allowing the user to easily access and filter data.

## Discussion

### Applications of SignaLink 2

Signaling cross-talks are important connections between different pathways and can generate novel input–output combinations as well as maintain the dynamic adaptation of the signaling system [39,40]. We have previously shown the significance of multi-pathway proteins (i.e., proteins functioning in more than one pathway) in the intertwined network of signaling pathways [1]. However, to ensure that an appropriate response is transduced, multi-pathway proteins need to be precisely regulated. SignaLink 2 contains multiple forms of protein regulation, including transcriptional, post-transcriptional and post-translational modifications. Thus, with SignaLink 2, regulation of each cross-talking protein can be analyzed.

Though cross-talk is generally defined as a physical interaction between pathway proteins, genetic studies often point out the importance of pathway cross-talks through transcription. In this case, cross-talk is mediated by a terminal transcription factor (TF) of a given pathway that regulates the expression of a component of another pathway [41,42]. With SignaLink 2, transcription-mediated pathway connections can be mapped as it contains i) uniformly defined pathways, ii) TFs for each pathway, and iii) a TF-regulatory network. Transcription-mediated cross-talks can be identified between any two pathways or globally at the systems-level. Recently, miRNAs have also been shown as important regulators of signaling pathways and networks [43,44]. As some miRNAs are known or predicted to be regulated by specific TFs [33,34], pathway cross-talks can be formed by a terminal TF of a pathway that regulates the expression of a miRNA down-regulating a component of another pathway. As an integrated database, SignaLink 2 contains TF and miRNA regulation data as well as a uniformly curated pathway dataset. These properties allow researchers to analyze pathway cross-talks on the post-transcriptional level. Furthermore, systems-level comparison of transcriptional, post-transcriptional and post-translational (i.e., PPI mediated) cross-talks can be performed with SignaLink 2.

Modeling signaling networks is a key approach to understand their dynamic properties in adaptation and diseases [45,46]. However, most PPI resources contain most interactions without direction (an information that is critical in signal transduction), pathway databases are often curated without uniform curation protocol and pathway definition, and generally lack important components having no direct signaling functions (i.e., scaffolds proteins, ubiquitin ligases and many phosphatases). As function of these components is the spatial and temporal regulation of the signaling flow, including them to a pathway resource could facilitate more precise modeling of signaling systems. As SignaLink 2 contains these components, it can enhance the development of models that can be successfully validated in wet lab experiments. Furthermore, data in SignaLink 2 is ready to use for modeling programs and scripts as users can download the files in well-known network and modeling formats (e.g., SMBL, BioPAX). User-specific selection of SignaLink 2 can be integrated with experimental data on enzyme activity or binding strength, thus, SignaLink 2 provides a general network topology for in-depth differential equitation modeling. Data from SignaLink 2 can be easily integrated to Boolean modeling frameworks, such as CellNetOptimizer [47]. Combining SignaLink 2 dataset (*i.e.,* signaling pathways and regulatory components) with network data of other cellular processes, such as autophagy or apoptosis, would allow Bayesian modeling on the regulation of these processes.

Genome programs and high-throughput screenings have greatly contributed to the construction of signaling networks in various model organisms, ranging from invertebrates to mammals. Reliable network resources enable the prediction of novel components and functions by analyzing cross-species data with the toolbox of functional genomics [48,49]. Accordingly, for *C. elegans*, *D. melanogaster* and *H. sapiens*, we have predicted 271 novel signaling components (i.e., signalogs) based on ortholog information of the previous version of SignaLink [50]. SignaLink 2 contains updated and extended dataset allowing the identification of further signalogs. In addition, SignaLink 2 enables the prediction of regulogs (i.e., predicted regulatory connections) as it contains TFs and regulatory connections for three metazoan species in a unified data structure [51].

Studying cross-talks, pathway regulator TFs and miRNAs have high pathological relevance as their malfunction often lead to diseases such as cancer [52]. Earlier, we found a significant change in the expression level of multi-pathway proteins in hepatocellular carcinoma [1], indicating that integration of signaling networks with expression datasets could reveal novel diagnostic and prognostic markers. The multilevel regulatory networks of SignaLink 2 have higher coverage and could serve as a more precise resource to compare normal and disease states of signaling networks.

Pharmacological targeting of key signaling components, including multi-pathway proteins and miRNAs is a promising strategy [53-55]. But unfortunately, numerous failures are known where the drug target had undiscovered or underestimated cross-talk as well as regulatory effects [56,57]. With SignaLink 2 different layers of signaling pathway regulation can be examined within a single resource. Performing an *in silico* perturbation analysis on the multi-layered signaling network of SignaLink 2 may facilitate the development of pharmacological interventions [54,58,59]. A perturbation analysis with SignaLink 2 can uncover key proteins or interactions important in the robustness of the signaling network. We have recently reviewed several such network perturbation approaches [60]. SignaLink 2 allows drug developers to measure the regulatory influence of a drug target candidate as well as to predict the signaling effect of its targeting. For example, drug targeting of a TF or its upstream interactor may influence the expression of many target genes, including signaling-related feedback mechanisms or metabolic enzymes important in drug metabolism. Applying the multi-layered network of SignaLink 2 could help developers to identify and avoid such circuits. SignaLink 2 can also support the identification of multiple targets in a multi-target pharmacological approach [53]: selecting the primarily suggested drug targets in the network of SignaLink 2 would allow the short listing of a minimal set of key targets with maximal impact on the network. Similarly, in anti-cancer strategy often not a single biochemical species is targeted but a complete pathway [60]. The stimulatory and inhibitory cross-talks and regulatory circuits of SignaLink 2 allow the listing of key regulators of a pathway, whose modulation can have pathway-level effects.

### Examples

First, we illustrate the usage of the http://SignaLink.org website with a protein, AXIN1. Next, the advantages of the download options of SignaLink 2 are illustrated with an integrated map of Notch and TGF-β pathways.

We selected AXIN1 to demonstrate how regulatory interactions can be examined for a given protein with the protein datasheet of the SignaLink 2 website. With the previous version of SignaLink, we found that AXIN1 is a multi-pathway protein having connection to more than one pathway [1]. Accordingly, AXIN1 was also described as a master scaffold for multiple signaling pathways [20]. Besides its distinct roles in the WNT, TGF-β and MAPK pathways, AXIN also has cross-talk functions [20]. Thus, one can think that AXIN1 should be precisely regulated. Here, we intended to explore how AXIN1 can be regulated to act as a multi-pathway protein and a master scaffold for members of different pathway. If we search for ‘AXIN1’ on the SignaLink 2 website, we will get its protein datasheet. On the top of the datasheet basic information about AXIN1 are presented (Figure 5a): 1) hyperlinks to AXIN1's page in other databases (Ensembl and UniProt); 2) AXIN1 is listed in SignaLink 2 as a mediator and a scaffold protein, and had been assigned to three pathways: RTK, WNT/Wingless and TGF. Below this box, the list of AXIN1 interactions can be found, grouped by layers (Figure 5b). In some cases the list is rather long, so each layer is expanded only if the user clicks on the layer’s title, and an optional sliding box on the left side of the page helps in navigation. In this view the names of interacting protein pairs, the type of interactions (coded with arrows) and pathway memberships are also visible. We can see that SignaLink 2 contains: 1) 15 interactions between AXIN and different pathway member proteins; 2) 8 interactions where AXIN as a scaffold regulates pathway proteins; 3) 136 predicted post-translational modifications (i.e., enzymes that may modify AXIN1); 4) 9 known PPIs predicted to be directed; 5) 17 predicted or known transcription factors that regulate AXIN1; 6) 28 predicted or known miRNAs that could down-regulate AXIN1; 7) Finally, 30 PPIs without any direction. Note that interactions can overlap between the layers, but these overlaps are mentioned for each relevant interaction. In certain cases, some overlaps can point out important feedbacks, showing a protein that interacts with and regulates AXIN1. Users can search or browse for each interactor/regulator of AXIN at the top of the list of layers. If we expand the first layer (Figure 5b), we can see that the first two interactions are direct stimulatory (normal arrow), the third one is indirect and inhibitory (dashed line with blunted arrow). To get more information about an interaction (for example, about the interaction between AXIN1 and PPP2CA), a simple click on the list is enough. Then, in the same box detailed information is shown (expanded in Figure 5c): the interaction between AXIN1 and PPP2CA was manually curated, two references to articles hyperlinked to PubMed can also be seen. In the list of sources, beside SignaLink, two integrated databases, BioGrid and HPRD, are also listed as having data about this interaction. At the bottom of this box, a GO semantic similarity score with a value of 0.53 is shown as a predicted level of confidence. The details of this score can be examined with a hyperlink to its original article. On the right side of the protein datasheet page, an interactive image of the network of first neighbors of AXIN1 takes place (Figure 5d). By default, in this network we can see that AXIN1 has 17 first neighbors among pathway members and scaffold-partners. Many of these neighbors are connected also to each other, e.g. APC2 and GSK3B, which form a feed-forward loop with AXIN1. Layers are color-coded, while interaction types are signed by arrow shapes. Different layers can be shown or hidden, and the network image can be switched to full screen mode easily with a control panel that also serves as a figure legend. To facilitate further exploration of the AXIN1 network, any click on the nodes of the network image will direct the user to the datasheet any protein or miRNA. In conclusion, the integrated regulatory data shown for AXIN1 in the SignaLink 2 website lists and points out molecular components, which are capable to regulate the expression or the function of AXIN1. As malfunction of AXIN1 is implicated in many diseases, including for example colon cancer [61], identification of AXIN1 regulators could serve as novel therapeutic targets. A short list of suggested – alternative – targets that could modulate AXIN1 could be important as currently there is no drug against AXIN1 (according to DrugBank and PharmGKB [62,63]). This strategy is in agreement with the recently proposed allo-network drug concept, whose effects can propagate across several proteins, to enhance or inhibit specific interactions along a pathway [64]. However, further experimental tests and global screens should clarify the tissue- and context-specific roles of these AXIN1 regulators, as well as their possible pharmacological applicability. We believe that SignaLink 2 can serve as an initial resource to identify such promising components.

**Figure 6:**
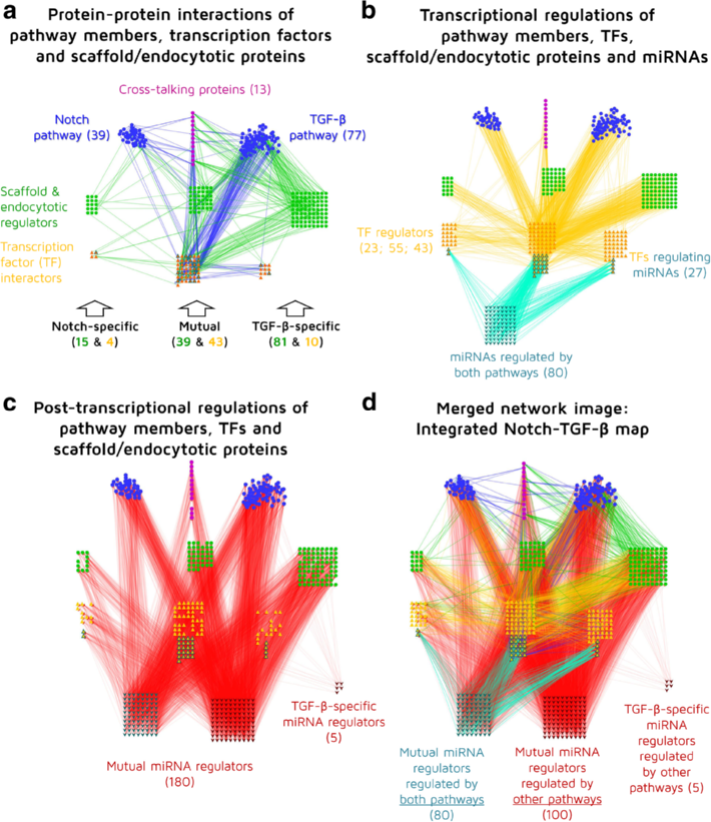
**Visualization of the multi-level cross-talk between Notch and TGF-β pathways to illustrate the capabilities of a SignaLink 2 download file.** In each part of the figure, text boxes show the color legend of the nodes. Number of components is listed in parentheses. **a**) Protein-protein interactions of Notch and TGF-β pathway members, transcription factors, scaffold and endocytotic proteins. Interactions between pathway members and of transcription factors (TFs) are shown with blue edges, while the interaction of scaffold and endocytotic proteins are shown with green edges. **b**) Transcriptional regulations of Notch and TGF-β pathway members, TFs, scaffold/endocytotic proteins and miRNAs. Transcriptional regulations of proteins and miRNAs are shown with orange and light blue edges, respectively. Those TFs that regulate miRNAs are highlighted with a light blue border. **c**) Post-transcriptional regulations of Notch and TGF-β pathway members, TFs and scaffold/endocytotic proteins. Post-transcriptional connection between miRNAs and their target proteins is shown with red edges. **d**) An integrated Notch – TGF-β map. This is a merged network image of the previous three networks. With textboxes we highlighted cross-talk regulating miRNAs. See the main text for the details of each network. The networks were analyzed and visualized with Cytoscape [69].

To illustrate the usage of SignaLink 2 download options, we selected two pathways, Notch and TGF-β. These two pathways have mostly different biochemical reactions and their members have distant evolutionary relations. Both pathways have been extensively studied as cross-talking pathways, having connections with other pathways [65,66]. Interestingly, in many cases Notch and TGF-β pathways have different functions, but in special cases they do cross-talk [67,68]. For a systems-level identification of these cross-talks, we need an integrated map of the Notch and TGF-β pathways. With SignaLink 2, this map can be generated, downloaded and visualized easily with Cytoscape [69]. On the Download page of SignaLink 2, we have selected both Notch and TGF-β pathways, their pathway regulators (scaffolds and endocytotic proteins), as well as their transcriptional and post-transcriptional regulators (i.e., TFs and miRNAs, respectively). (We have not included other layers to focus on these three groups.) We have chosen the Cytoscape format, which allowed us to start working with the network right after we have downloaded it. Note that the SQL query behind this download is quite complex, thus, generation of the Cytoscape file needs time. When we opened the Cytoscape file, we used the attributes already implemented for the nodes and edges of the network. Thus, no further data imports were necessary. We grouped the components to Notch, TGF-β and mutual groups, and for the sake of visibility and simplicity, we excluded those components that belonged to other pathways. Components of other pathways were originally included in the file as they had cross-talks with Notch and/or TGF-β pathway components. Then, using the filtering and network layout options of Cytoscape, we have created three images showing the protein-protein, transcriptional and post-transcriptional regulatory interactions of the two pathways. On Figure 6a, we can see the members and the interactions of Notch and TGF-β pathway proteins (blue nodes and edges), their scaffold and endocytotic proteins (green nodes and edges). Transcription factors of the pathways are highlighted with orange. This image shows that the pathways have 13 cross-talking proteins that bridge the pathways. Both pathways have specific and also mutual scaffolds and target TF. Note that these TFs are not regulators but the terminal components of the pathways. All the protein numbers are shown in parentheses. On Figure 6b, the transcriptional regulation of the two pathways is visualized. For the sake of clarity, we only visualized experimentally verified transcriptional interactions. This filtering can be done either before downloading the file using the Advanced Filter option of SignaLink 2, or after the download by using the Filter option of Cytoscape as all interactions can be filtered by their source. In this network image, TFs that regulate the expression of the pathway members and the scaffold/endocytotic components are listed and grouped by their pathway specificity. The high number of mutual TFs indicates a regulated co-expression of Notch and TGF-β pathway members. This is in agreement with the earlier finding that Notch and TGF-β pathways have a high-level of cross-talks [67,68] (i.e., co-expression of cross-talking proteins is essential to form cross-talks). Interestingly, some of the terminal TFs, already listed in Figure 6a, regulate miRNAs and not proteins. These TFs have a light blue border and they have a cyan colored edge to their target miRNAs (shown with light blue nodes). These miRNAs are regulated by the pathways. Note that in our analysis we found miRNAs that are regulated by both pathways and not specifically by one of the pathways. These 80 mutual miRNAs can be interpreted as another example for the high-level cooperation between the Notch and TGF-β pathways. However, it is important to note that we have data on the miRNA regulation role for only a subset of TFs. Thus, upon new TF-miRNA regulatory interactions will be described, we may be able to identify miRNAs regulated by only one pathway. On Figure 6c, we show the post-transcriptional regulations of the already listed proteins. The miRNAs that could down-regulate a protein are shown with dark red having red colored interactions. For visibility reasons, we have excluded the high number of predicted interactions retrieved from TargetScan. We found 5 miRNAs that specifically target TGF-β pathway components. Compared to the mutual 180 miRNAs, these miRNAs could serve as specific regulators of the TGF-β pathways, while not affecting the Notch pathway. The merged network image in Figure 6d presents the integrated map of Notch and TGF-β pathways. For the mutual miRNAs we could identify two distinct sets: We found 80 miRNAs regulated by both pathways that are capable to regulate members of both pathways, while 100 miRNAs can regulate members of both pathways but they are regulated by other pathways. The 80 miRNAs can be considered as mediators of post-transcriptional cross-talks between Notch and TGF-β pathways. Consequently, miRNAs regulated by other pathways, but targeting TGF-β or TGF-β and Notch pathway members could be the possible mediators of further pathway cross-talks on the post-transcriptional level. Recently, the role of miRNA in the regulation of Notch, TGF-β and other pathways has been highlighted in hepatocellular carcinoma and cancer stem cells [70]. We believe that SignaLink 2 could serve as a systems-level resource to list pathway-specific and mutual miRNAs as well as cross-talks on the post-transcriptional level. This list may guide further experimental studies to compare and test the role of these miRNAs in pathway functions. In conclusion, using SignaLink 2 data with Cytoscape, we could generate and visualize a multi-level cross-talk network of the Notch and TGF-β pathways, identify mutual and pathway-specific components/regulators, as well as point out important TFs and miRNAs that could regulate cross-talk between these two pathways and other pathways. We note that many of these interactions may be highly context-specific and experimental validation is needed to confirm the function of each inter-pathway connection.

### Comparison with other resources

Several excellent resources already exist that contain similar information as SignaLink 2. In Table 2 we compare the major features of SignaLink 2 with three well-known pathway databases, with two integrated resources and with the first version of SignaLink. We note that one of the resources, ConsensusPathDB, contains selected data from SignaLink. All of these resources contain valuable data but none of them have all the features and data types as SignaLink 2 does. Furthermore, as compared to other signaling resources, SignaLink 2 has the following unique features: 1) it contains experimental data not only from humans but for two invertebrate model organisms; 2) combines large-scale datasets with manual curation; 3) provides confidence scores for each interaction; 4) operates a highly customizable download page with multiple file formats. Most resources contain different data types in one database, making their selection difficult. According to our comparison, SignaLink 2 was the only resource that lists the transcriptional, post-transcriptional and post-translational regulators of a pathway. In SignaLink 2, these data types are stored in a multi-layered structure allowing users a simple way to analyze the layers of interest in the signaling network. We acknowledge that SignaLink 2 contains data for seven pathways, while some resources (e.g., KEGG, Reactome) contain significantly more pathways. As pathways in SignaLink 2 encompass major developmental and important disease-related pathways, we believe that the overall information it can provide fills a gap and supports a more detailed analysis of these pathways.

**Table 2:** Comparison of resources with SignaLink 2

	**KEGG**[71]	**Reactome**[72]	**SPIKE**[73]	**ConsensusPathDB**[74]	**TranscriptomeBrowser**[10]	**SignaLink 1**[1]	**SignaLink 2**
**Model species**^**#**^				√		√	√
**Containsmanual curation**^**§**^	√	√	√			√	√
**Contains integrated data**				√	√		√
**Reference for each interaction**		√	√			√	√
Cross-talks and multi-pathway proteins^*^		√				√	√
**Undirected protein-protein interactions**	√	√		√	√		√
**Directed protein-protein interactions**^**†**^					√		√
**Transcription factor regulation**		√		√	√		√
**miRNA regulation**					√		√
**Transcription factors that regulate miRNAs**							√
**Confidence score for the interactions**							√
**Customizable download options**					√^ˣ^	√	√
**Freely downloadable for academic users**		√	√	√	√	√	√

We compared the major features of SignaLink 2 with three pathway databases (KEGG, Reactome and SPIKE), an integrated pathway resource, ConsensusPathDB, and an integrated regulatory resource, TranscriptomeBrowser. We also compared the features of SignaLink 2 with its previous version to show the major upgrade and extension. ^#^Only those species were taken into account that were represented with experimental data in the resource (i.e., orthology derived predictions were not considered). ^§^ Manual curation performed by the developers of the resource (i.e., not integrated from external resources). ^*^ All types of cross-talks (transcriptional, post-transcriptional and post-translational) were taken into account but only in those cases where the structure of the resource allowed to easily download cross-talks and multi-pathway proteins (i.e., without any computational evaluation). ^**†**^ Only protein-protein interactions (PPIs) with predicted directions were taken into account. ˣ Only available with the InteractomeBrowser plugin.

We previously compared the number of proteins and interactions between SignaLink 1, KEGG, Reactome and NetPath [1]. Since this comparison only the datasets of SignaLink and Reactome has improved significantly, thus, here we present an updated comparison between SignaLink 2 and Reactome. We compared the proteins and interactions of five pathways (JAK/STAT, Notch, RTK, TGF-β and WNT) present in both SignaLink 2 and Reactome. We found that 331 proteins and 848 interactions were present in both resources. 677 proteins and 5,962 interactions were SignaLink-specific, while 916 proteins and 5,365 interactions were Reactome-specific. Previously we also compared the curation strategies of SignaLink and Reactome and suggested the high number of protein complex based interactions in Reactome and the significant bias towards specific enzyme functions (*e.g.,* proteolysis) as a possible explanation of the high number resource-specific proteins and interactions [1]. Overall, we think that SignaLink 2 both complements the datasets found in other databases, such as Reactome and supports a more detailed analysis of these pathways by including transcriptional, post-transcriptional and possible post-translational regulators of a pathway.

### Future plans

Knowing that the list of components and interactions in each layer is not complete, we will include further experimentally validated datasets yearly, which will complement the manual curation update we perform every two years. We also intend to include cellular compartment and tissue-specific localization information to future versions of SignaLink. In addition, we will increase the number of integrated hyperlinks in the protein datasheet page and develop connections to medical and drug-related resources.

## Conclusions

We presented the upgraded and extended version of the SignaLink resource that allows users to explore signaling pathway interactions and to identify pathway regulators, as well as transcriptional and post-transcriptional regulatory components. With SignaLink 2 users can examine in a single resource how scaffolds, enzymes, TFs or miRNAs regulate cross-talks and signaling flow. We hope that SignaLink 2 will be an efficient resource for modeling signaling systems as well as for signaling-related network medicine and pharmacology.

### Availability and requirements

Non-profit users can access SignaLink 2 free of charge at http://SignaLink.org.

## Competing interests

The authors declare no competing interests.

## Authors’ contributions

MK, ZD and MP performed the manual curation. DF, DT, DM and MSB integrated the external resources and constructed the SignaLink 2 database. DT developed the website, DF wrote the download module. LZ, KL, FIJ and TK participated in the design of the database and website. TK coordinated the work. FIJ, TV, PC and TK wrote the manuscript. All authors read and approved the final manuscript.
